# Effect of endurance exercise duration on muscle hypertrophy induced by functional overload

**DOI:** 10.1002/2211-5463.13028

**Published:** 2020-12-08

**Authors:** Takanaga Shirai, Tsubasa Obara, Tohru Takemasa

**Affiliations:** ^1^ Graduate School of Comprehensive Human Sciences University of Tsukuba Japan; ^2^ Japan Society for the Promotion of Science Tokyo Japan; ^3^ School of Physical Education, Health and Sport Sciences Tsukuba Japan; ^4^ Yokohama B—Corsairs Co., Ltd. Japan; ^5^ Faculty of Health and Sport Sciences University of Tsukuba Japan

**Keywords:** endurance training, exercise duration, hypertrophy, mTOR signaling, protein breakdown, resistance training

## Abstract

For many ball games, both resistance and endurance training are necessary to improve muscle strength and endurance capacity. Endurance training has been reported to inhibit muscle strength and hypertrophy, but some studies have reported that endurance exercise (EE) does not inhibit the effects of resistance exercise. Here, we examined the effect of short‐ or long‐duration EE on mouse skeletal muscle hypertrophy induced by functional overload (OL) at the molecular level. Plantaris muscle hypertrophy was induced by OL with synergist ablation in mice. Body mass was reduced with endurance training, but EE duration (30 or 90 min) had no effect. The ratio of plantaris muscle weight to body weight was higher in the OL and EE for 30 min (OL+EE30) and OL and EE for 90 min (OL+EE90) groups compared with the OL group. Expression of mechanistic target of rapamycin signaling proteins, which is related to protein synthesis and hypertrophy, was increased in the OL+EE30 group. Expression of Forkhead box‐containing protein O1, which is related to protein breakdown and atrophy, remained unchanged. However, microtubule‐associated protein 1 light chain 3, a known marker of autophagy, and MAFbx, which is related to protein breakdown, were significantly increased in the OL+EE90 group. Furthermore, markers of oxidative stress, ubiquitin and 4‐hydroxynonenal were also significantly increased in the OL+EE90 group compared with other groups. In conclusion, EE duration did not affect body mass and plantaris mass and did not interfere with mechanistic target of rapamycin signaling, but it did increase ubiquitinated proteins and oxidative stress. It is therefore necessary to consider training durations for EE when combining endurance and resistance training.

## Graphical abstract

Functional overload is known as a model for inducing muscle hypertrophy in rodents. It has been reported that endurance exercise (EE) suppresses muscle hypertrophy, and we investigated the effect of EE time on hypertrophied muscles. Long‐duration EE during functional overload does not suppress muscle hypertrophy but causes increased protein degradation and oxidative stress.

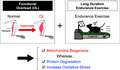

**Abbreviations**
4E‐BP1: 4E‐binding protein 1
4HNE: 4‐hydroxynonenal
Akt: protein kinase B
AMPK: AMP‐activated protein kinase
EE: endurance exercise
ERK1/2: extracellular signal‐regulated kinases 1/2
FoxO1: Forkhead box‐containing protein O1
GSK3β: glycogen synthase kinase 3β
LC‐3: microtubule‐associated protein 1 light chain 3
MAFbx: Muscle Atrophy F‐box
MAPK: mitogen‐activated protein kinase
mTOR: mechanistic target of rapamycin
MuRF1: Muscle RING‐Finger Protein
OL: overload
OL+EE30: OL and EE for 30 min
OL+EE90: OL and EE for 90 min
p70S6K: p70 S6 kinase
PGC‐1α: peroxisome proliferator‐activated receptor γ coactivator‐1α
PI3K: phosphoinositide 3‐kinase
S6: S6 ribosomal protein S6
VEGF: vascular endothelial growth factor

Athletes endure various training regimens on a daily basis to improve their competitive capacity. Training is mainly classified as resistance or endurance training, with differing effects depending on exercise type, duration, intensity and frequency. Consequently, athletes need to carefully match the training with their respective objectives [1, 2]. Resistance training mainly changes the morphology of skeletal muscle, improves muscle strength and promotes hypertrophy [3, 4], and induces muscle hypertrophy by activating the mammalian/mechanistic target of rapamycin (mTOR) signaling pathway, which contains p70 S6 kinase (p70S6K) and S6 ribosomal protein (S6) [5]. In addition, mitogen‐activated protein kinase (MAPK) signaling is activated by a variety of cellular stresses, including mechanical stress. Muscle contraction caused by resistance exercise is also known to activate extracellular signal‐regulated kinases 1/2 (ERK1/2), p38 MAPK and c‐Jun N‐terminal kinase, members of the MAPK family [6, 7]. In contrast, endurance training mainly changes the function of skeletal muscle and improves metabolic ability [8, 9] and activates AMP‐activated protein kinase (AMPK) by way of AMP produced by adenylate kinase [10]. Consequently, peroxisome proliferator‐activated receptor γ coactivator‐1α (PGC‐1α), a master regulator of energy metabolism and mitochondrial biogenesis, is activated, and the metabolic capacity is improved [11].

In competitive sports, training is performed according to their characteristics. For many ball games, both resistance and endurance training are necessary to improve muscle strength and endurance capacity. In previous studies, endurance training was reported to inhibit muscle strength and hypertrophy [12]. This phenomenon is called the “interference effect” [13, 14]. For these reasons, AMPK interferes with mTOR signaling via tuberous sclerosis complex 2 and is thought to suppress protein synthesis [15]. Conversely, some papers have also reported that endurance exercise (EE) did not inhibit the effects of resistance exercise [13, 16]. No consensus regarding the influence of endurance training on resistance training has been obtained until now [17].

We considered a training protocol that minimizes interference effects by monitoring the activation levels of molecules involved in muscle adaptation through scientific investigations of molecular exercise physiology [18]. In fact, few investigations have focused on the effect of endurance training duration on muscle hypertrophy at a molecular level. Therefore, the aim of this study was to examine the effect of short‐ or long‐duration EE on mouse skeletal muscle hypertrophy induced by functional overload (OL) at the molecular level. We hypothesized that the effects of resistance exercise would be suppressed by long‐duration EE, and that long‐duration EE restricted muscle hypertrophy.

## Materials and methods

### Experimental approval

Animal experiments were carried out in a humane manner after receiving approval from the Institutional Animal Experiment Committee of the University of Tsukuba and in accordance with the Regulations for Animal Experimentation of the university and the fundamental Guidelines for Proper Conduct of Animal Experiments and Related Activities in Academic Research Institutions under the jurisdiction of the Ministry of Education, Culture, Sports, Science, and Technology of Japan. All experimental procedures performed in this study were approved by the Institutional Animal Experiment Committee of the University of Tsukuba (15‐057).

### Animals

Male ICR mice aged 7–8 weeks (Tokyo Laboratory Animals Science Co., Tokyo, Japan) were used in this study. Mice were kept in temperature‐ (22 ± 2 °C) and humidity (55% ± 5%)‐controlled facilities under a 12/12‐h light/dark cycle with *ad libitum* access to food and water. On completion of experimental treatments, the mice were euthanized by cervical dislocation. Their lower limb muscles were then dissected 24 h after the last exercise session, weighed quickly, frozen in liquid nitrogen and stored at −80 °C until needed for analysis.

### Synergist ablation surgery

We performed lateral synergist ablation surgeries, as previously described, under anesthesia with 2.0% isoflurane air inhalation [19, 20]. This *in vivo* model induces hypertrophy of the plantaris muscle by mechanical OL through the surgical removal of synergist muscles (gastrocnemius and soleus). After 14 days, the mice were anesthetized, and the plantaris muscle was excised, weighed, quickly frozen in liquid nitrogen and stored at −80 °C.

### Endurance training

**Fig. 1:**
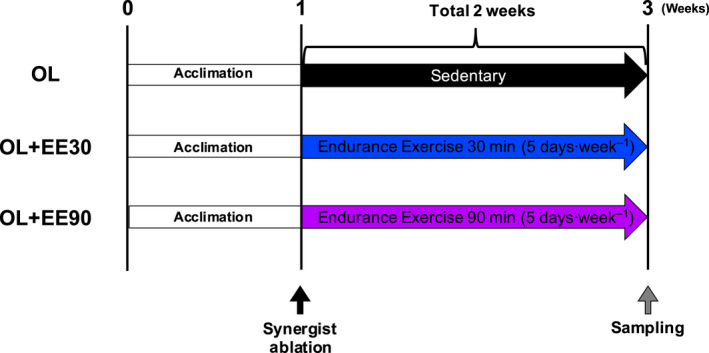
Experimental design. Mice were divided into three groups: nonexercise OL group, OL+EE30 group and OL+EE90 group. After 1‐week acclimation, the plantaris muscle was overloaded through synergist unilateral ablation. From day 1 after synergist ablation, mice were placed on a flat treadmill to run for 30 or 90 min at a speed of 20 m·min^−1^ (5 days·week^−1^) for 2 weeks. *n* = 7–8 per group.

The experimental design is shown in Fig. 1. Mice were familiarized with running on a rodent treadmill, at 10–20 m·min^−1^, for 3 days before the experiment. On day 1 following synergist ablation, they were placed on a flat treadmill for 30 or 90 min at a speed of 20 m·min^−1^ (5 days·week^−1^) for 2 weeks. We then evaluated the effect of duration of EE. The animals were randomly divided into three groups: nonexercise OL group, OL and EE for 30 min (OL+EE30) group, and OL and EE for 90 min (OL+EE90) group.

### Western blotting

Excised plantaris muscles were immediately frozen in liquid nitrogen, and total muscle protein was extracted by lysis buffer containing 50 mm HEPES (pH 7.6), 150 mm NaCl, 10 mm EDTA, 10 mm Na_4_P_2_O_7_, 10 mm NaF, 2 mm Na_3_VO_4_, 1% (v/v) Nonidet P‐40, 1% (v/v) Na‐deoxycholate, 0.2% (w/v) SDS and 1% (v/v) complete protease inhibitor cocktail. Protein concentrations were measured using a Protein Assay Bicinchoninate Kit (Nacalai Tesque Inc., Kyoto, Japan). Before SDS/PAGE, an aliquot of the extracted protein solution was mixed with equal volumes sample loading buffer containing 1% (v/v) 2‐mercaptoethanol, 4% (w/v) SDS, 125 mm Tris–HCl (pH 6.8), 10% (w/v) sucrose and 0.01% (w/v) bromophenol blue. The mixture was then heated at 97 °C for 3 min. Ten micrograms of protein was separated on an SDS/polyacrylamide gel and electrically transferred to an ImmunoBlot poly(vinylidene difluoride) membrane (Bio‐Rad Laboratories, Hercules, CA, USA). The blot was blocked by Blocking One (Nakalai Tesque Inc.) for 1 h at room temperature and incubated with primary antibodies overnight at 4 °C in TBS containing 0.1% Tween 20. Signals were detected using the Immunostar Zeta or LD (Wako Chemicals, Osaka, Japan), quantified by C‐Digit (LI‐COR Biosciences, Lincoln, NE, USA) and expressed as arbitrary units. The expression levels of each protein were normalized to that of glyceraldehyde‐3‐phosphate dehydrogenase.

### Primary antibodies for western blotting

The following primary antibodies were used for western blotting: protein kinase B (Akt) (9272; Cell Signaling Technology, Danvers, MA, USA), p‐Akt (#4060S; Cell Signaling Technology), p70S6K (#9202; Cell Signaling Technology), p‐p70S6K (#9205; Cell Signaling Technology), S6 ribosomal protein S6 (S6) (#2217; Cell Signaling Technology), p‐S6 (#4858S; Cell Signaling Technology), 4E‐binding protein 1 (4E‐BP1) (#9452; Cell Signaling Technology), p‐4E‐BP1 (#9459; Cell Signaling Technology), glycogen synthase kinase 3β (GSK3β) (#12456; Cell Signaling Technology), p‐GSK3β (#5558; Cell Signaling Technology), ERK1/2 (#4695; Cell Signaling Technology), p‐ERK1/2 (#4370; Cell Signaling Technology), p38 (#9212; Cell Signaling Technology), p‐p38 (#9211; Cell Signaling Technology), AMPK (#2532; Cell Signaling Technology), p‐AMPK (#2531; Cell Signaling Technology), ATP synthase (sc‐517267; Santa Cruz Biotechnology, Santa Cruz, CA, USA), p‐ATP synthase (sc‐374647; Santa Cruz Biotechnology), PGC‐1α (516557; Millipore, Burlington, MA, USA) and cytochrome *c* (556433; BD Biosciences, San Jose, CA, USA), vascular endothelial growth factor (VEGF) (sc‐507; Santa Cruz Biotechnology), oxidative phosphorylation (ab110413; Abcam, Cambridge, UK), glyceraldehyde‐3‐phosphate dehydrogenase (ab8245; Abcam), Forkhead box‐containing protein O1 (FoxO1) (2880S; Cell Signaling Technology), p‐FoxO1 (9461; Cell Signaling Technology), microtubule‐associated protein 1 light chain 3 (MAP1LC3; LC‐3) (#4108; Cell Signaling Technology), p62 (SQSTM1) (PM045; MBL), Muscle RING‐Finger Protein (MuRF1) (sc‐32920; Santa Cruz Biotechnology), Muscle Atrophy F‐box (MAFbx) (sc‐33782; Santa Cruz Biotechnology), 4‐hydroxy‐2‐nonenal (4‐HNE) (ab48506; Abcam) and ubiquitin (sc‐166553; Santa Cruz).

### Statistical analysis

Data are shown as means ± standard error. One‐way ANOVAs were conducted for all measurements. When a significant *P* value was obtained, statistical significance was calculated according to Tukey’s method. The graphpad prism 7 software (GraphPad, Inc., San Diego, CA, USA) was used for all statistical calculations, and the significance level was set to *P* < 0.05 for all cases.

## Results

### Body and skeletal muscle wet weight

**Fig. 2:**
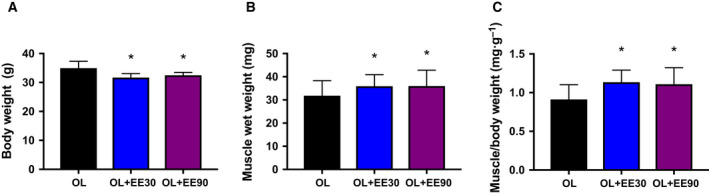
Body and plantaris wet weight. Body weight (A), muscle wet weight (B) and muscle/body weight (C). Values represent mean + standard error (*n* = 7–8 per group). *P < 0.05 versus OL by one‐way ANOVA.

To examine the effect of EE duration on hypertrophying muscle, we measured body mass and plantaris muscle mass. Endurance training decreased body mass but with no significant difference in both OL+EE30 and OL+EE90 groups (OL: 34.94 ± 0.84 g versus OL+EE30: 31.66 ± 0.53 g and OL+EE90: 32.49 ± 0.37 g) (Fig. 2A). Plantaris and body weight were increased in two groups with mice that underwent both synergist ablation and EE (OL: 100% ± 0.05% versus OL+EE30: 112% ± 0.04% and OL+EE90: 113% ± 0.05%) (Fig. 2B,C).

### Expression of mTOR signaling proteins

**Fig. 3:**
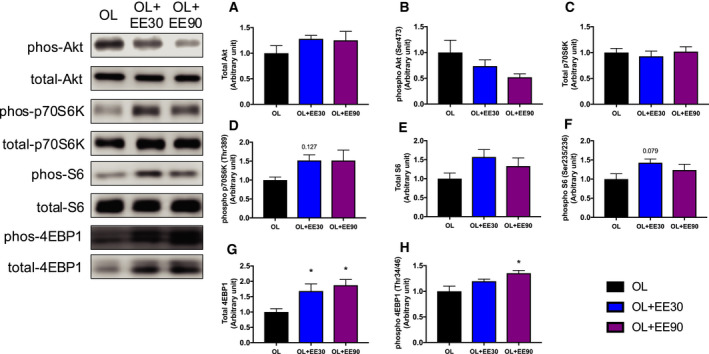
Effect of short‐ and long‐duration endurance training on mTOR signaling in hypertrophying skeletal muscle. Total Akt (A), phospho‐Akt (B), total p70S6K (C), phospho‐p70S6K (D), total S6 (E), phospho‐S6 (F), total 4EBP1 (G) and phospho‐4EBP1 (H) in the plantaris muscle. Values represent mean + standard error (*n* = 7–8 per group). *P < 0.05 versus OL by one‐way ANOVA.

We measured both the total and the phosphorylation levels of mTOR signaling molecules to investigate the effects of endurance training on hypertrophying muscles. After 2 weeks of endurance training, no significant difference in Akt, p70S6K and S6 (Fig. 3A–F) was observed. In addition, total and phosphorylation levels of 4E‐BP1 increased significantly in the OL+EE90 group compared with the OL group (total protein, OL: 100% ± 0.001% versus OL+EE90: 187% ± 0.18%; phospho, OL: 100% ± 0.001% versus OL+EE90: 135% ± 0.05%) (Fig. 3G,H).

### Expression of other signaling pathway

**Fig. 4:**
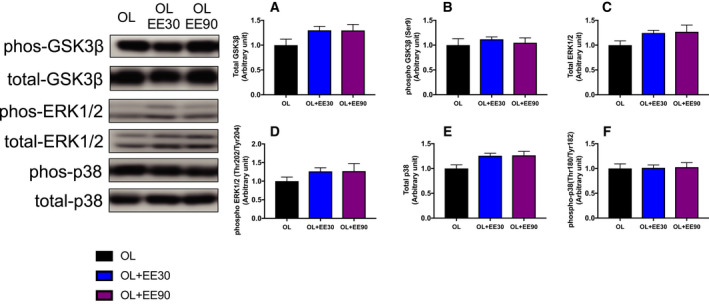
Effect of short‐ and long‐duration endurance training on MAPK signaling in hypertrophying skeletal muscle: total GSK3β (A), phospho‐GSK3β (B), total ERK1/2 (C), phospho‐ERK1/2 (D), total p38 (E) and phospho‐p38 (F).

Next, we also measured MAPK signaling, which is known to be an upstream regulator of mTOR and is activated by a variety of cellular stresses, including mechanical stress. However, there was no difference in MAPK signaling protein in each group (Fig. 4).

### Expression of oxidative metabolism‐related proteins and the mitochondria electron transport chain

**Fig. 5:**
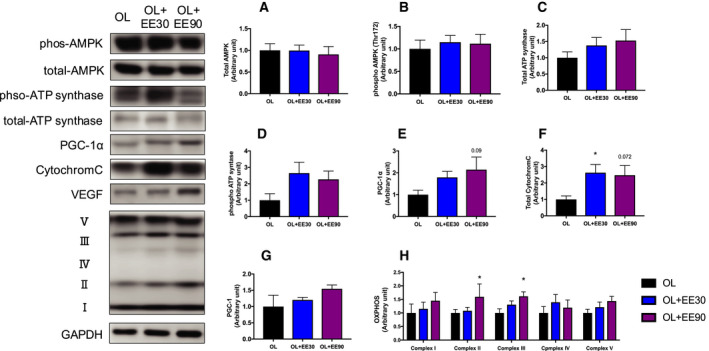
Effect of short‐ and long‐duration endurance training on metabolism and mitochondrial complex protein expression in hypertrophied skeletal muscle. Total AMPK (A), phospho‐AMPK (B), total ATP synthase (C), phospho‐ATP synthase (D), PGC‐1α (E), cytochrome *c* (F), VEGF (G) and oxidative phosphorylation (OXPHOS) (H) in the plantaris muscle. Values represent mean + standard error (*n* = 7–8 per group). *P < 0.05 versus OL by one‐way ANOVA. GAPDH, glyceraldehyde‐3‐phosphate dehydrogenase.

Significant differences in the expression levels of proteins related to oxidative metabolism (AMPK, ATP synthase) were not observed between groups (Fig. 5A–D). The protein expression levels of PGC‐1α, a known master regulator of metabolism, were increased, but this change was not significant (Fig. 5E). The cytochrome *c* expression levels were significantly increased in the OL+EE30 group compared with the OL group (OL: 100% ± 0.002% versus OL+EE30: 2.47% ± 0.59%) (Fig. 5F). VEGF, which is related to angiogenesis, showed no difference between the groups (Fig. 5G). The mitochondrial complex chains II and III were significantly increased in the OL+EE90 group compared with the OL group (complex chain II, OL: 100% ± 0.001% versus OL+E90: 159% ± 0.47%; complex chain III, OL: 100% ± 0.01% versus OL+EE90: 161% ± 0.16%) (Fig. 5H).

### Expression of ubiquitinated and oxidative stress proteins

**Fig. 6:**
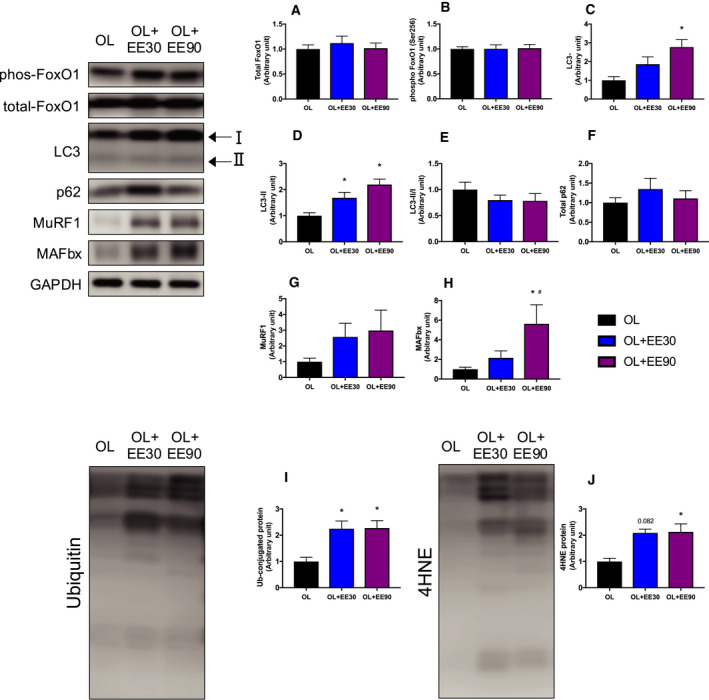
Effect of short‐ and long‐duration endurance training on catabolism signaling in hypertrophied skeletal muscle. Total FoxO1 (A), phospho‐FoxO1 (B), LC3‐I (C), LC3‐II (D), LC3‐II/I (E), p62 (F), MuRF1 (G), MAFbx (H), ubiquitin (I) and 4‐hydroxynonenal (4HNE) (J) in the plantaris muscle. Values represent mean + standard error (*n* = 7–8 per group). *P < 0.05 versus OL by one‐way ANOVA.

Protein degradation in skeletal muscle, through both the ubiquitin–proteasome pathway and autophagy lysosomes, could potentially have been enhanced by decreased phosphoinositide 3‐kinase (PI3K)/Akt signaling [21, 22]. We measured the expression levels of these catabolic proteins in all three groups. After training for 2 weeks, there was no significant change in the FoxO1 expression levels (Fig. 6A,B). Conversely, LC3, a marker of autophagy, was significantly increased in the OL+EE90 group compared with the OL group (OL: 100% ± 0.01% versus OL+EE90: 2.77% ± 0.39%) (Fig. 6C,D). The LC3II/I ratio remained unchanged for each group (Fig. 6E). The p62 expression levels, related to autophagy, were not significantly changed in any group (Fig. 6F). MuRF1 downstream of PI3K/Akt signaling showed no difference between the groups, but MAFbx were significantly higher in the OL+EE90 group compared with the OL and OL+EE30 groups (Fig. 6G,H). Ubiquitin expression was significantly increased in the OL+EE30 and OL+EE90 groups compared with the OL group (OL: 100% ± 0.01% versus OL+EE30: 224% ± 0.29% and OL+EE90: 227% ± 0.28%) (Fig. 6I). A marker of oxidative stress, 4‐HNE, was significantly increased in the OL+EE90 group compared with the OL group (OL: 100% ± 0.01% versus OL+EE90: 212% ± 0.3%) (Fig. 6J).

## Discussion

In this study on the effects of duration of EE on muscle hypertrophy induced by functional OL, we demonstrated that short‐duration EE increased mTOR signaling activation, and long‐duration EE increased oxidative stress or protein and lipid modifications (ubiquitin and 4‐hydroxynonenal) expression in mouse skeletal muscle tissues. After 2 weeks of endurance training, we observed body weight loss in the OL+EE30 and OL+EE90 groups compared with the OL group. Previous human studies reported body weight increases in resistance‐trained groups, with no changes for the endurance‐trained group [12]. Here, a similar change was observed using mouse models.

A previous investigation reported suppressed muscle hypertrophy in the group that performed both resistance and endurance training compared with the resistance training‐only group [23]. Therefore, we hypothesized that muscle hypertrophy is suppressed by performing endurance training during functional OL. In this study, the plantaris wet weight per body weight increased in the OL+EE30 and OL+EE90 groups. This suggested that endurance training for 30–90 min did not suppress the increase of muscle wet weight through functional OL. As a limitation, our experiments did not include the control group (sham operation) and therefore could not provide evidence of causing muscle hypertrophy in each mouse. However, we demonstrated that the muscle wet weight was similar to that of the OL group in the previous study using the same protocol [24, 25], and the OL used in this experiment caused muscle hypertrophy.

We then validated the effects of EE on mTOR signaling by examining signal molecules in skeletal muscle tissue. Akt expression levels did not differ significantly between groups. Previous studies have shown that endurance training increased total Akt expression [26]. In a rat study, Akt phosphorylation was increased by both resistance and endurance training, but with no difference when considering the order of resistance and aerobic training [27]. In this study, the duration of 30‐ or 90‐min endurance training did not alter Akt expression. In this study, duration of endurance training for 30 or 90 min did not alter the protein expression of Akt during functional OL.

It is known that p70S6K, a downstream effector of the muscle hypertrophy signal, is phosphorylated by resistance training [28]. In human studies, Fyfe *et al*. [29] reported reduced p70S6K phosphorylation in the concurrent training group compared with resistance training alone. Another investigation reported no significant difference in the expression levels of p70S6K between concurrent training groups and resistance training only [30]. In our study, p70S6K expression levels increased in the OL+EE30 group compared with the OL group. These results suggested that 30‐min EE activated the muscle hypertrophy response. However, no significant difference was observed in the p70S6K expression levels between the OL and OL+EE90 groups, suggesting that long‐duration EE did not promote the muscle hypertrophy response. From this, it can be considered that short‐duration EE may contribute to muscle hypertrophy, but long‐duration EE may have an adverse effect. The S6 expression levels showed no significant difference between groups. Previous human studies reported no significant difference between the resistance exercise group and combining resistance and high‐intensity interval exercise simultaneously [29]. Similar results were obtained in this study. One of the limitations of this study is that the efficiency of muscle protein synthesis using the SUnSET method has not been measured, and it is necessary to consider a detailed study in future studies.

Next, we examined the protein expression related to metabolism. For AMPK expression, a signal central to aerobic metabolism, we detected no difference in expression levels for the duration of endurance training. Previous studies on rat models reported that endurance and resistance training with electrical stimulation increased AMPK expression levels immediately after exercise, regardless of the exercise model, but returned to baseline 1 h later [1]. In this study, we assumed that increased AMPK expression as a result of EE returned to baseline, because sampling took place 1 h after the final EE. In addition, because functional OL always leads to mechanical stress, it was assumed that AMPK was phosphorylated in all groups; therefore, no significant difference was demonstrated between groups. In future investigations, longer exercise protocols may induce significantly increased AMPK.

We also quantified the expression of mitochondrial‐related proteins. PGC‐1α is a known metabolic master gene that is central to metabolism [1]. Previous studies have shown that PGC‐1α regulates mitochondrial protein expression [31]. In this study, the expression of mitochondrial respiratory chain complexes II and III was increased in the OL+EE90 group compared with the OL group. Previous studies have reported that electrical stimulation increased respiratory chain complex expression [32]. Furthermore, it has been reported that prolonged EE also increased respiratory chain complex expression. Therefore, respiratory chain complex protein expression may have increased in our study, as shown in previous investigations.

We also quantified catabolism protein expression to examine the effects on protein breakdown. FoxO1, a protein in the ubiquitin–proteasome system, remained unchanged during this investigation. FoxO1 is regulated downstream of Akt, and increased phosphorylation is known to inhibit FoxO1 translocation into the nucleus, which suppresses muscle atrophy [33]. In this investigation, Akt‐FoxO signals were not affected because Akt expression levels remained unchanged between groups. Because ubiquitin expression levels increased regardless of the duration of EE, it is presumed that unnecessary protein expression increased during EE.

We also quantified the proteins involved in the autophagy–lysosomal system. We examined LC‐3, a marker for autophagosomes, and p62, a marker for ubiquitinated proteins [34]. As a result, LC‐3 expression was significantly increased in the EE+90 group compared with the OL group. LC3 is known to be increased by prolonged exercise, and our results supported this finding. However, the LC3II/I ratio, which is a parameter of autophagy, remained unchanged for all groups. Although LC3 expression levels were increased by EE, there was no relative difference when expressed as a ratio. Next, the expression level of 4‐HNE, a marker for oxidative stress [35, 36], increased in the OL+EE90 group. Oxidative stress is known to increase during EE. In this investigation, mechanical stress caused by EE in addition to functional OL was shown to promote increased oxidative stress in the OL+EE90 group. Our data suggest that long‐duration EE increases ubiquitinated proteins and oxidative stress.

In this study, we examined the effects of prolonged EE on muscle hypertrophy. Many previous human studies that combined resistance training with endurance training demonstrated that endurance training had a negative effect on muscular hypertrophy, strength and power. However, this study demonstrated that short‐duration EE promoted the muscle hypertrophy response, whereas long‐duration EE suppressed the muscle hypertrophy response. In this experimental protocol, the EE time was 30 or 90 min (five times/week). However, the molecular mechanism by which EE inhibits muscle hypertrophy will be clarified only by longer‐duration EEs in the future. In addition, the effects on muscle morphology (fiber size, muscle fiber type, etc.) should also be addressed in future research. Because the molecular response changes in relation to sampling time, an experiment with several sampling points should be considered. When combining endurance and resistance exercises, it is necessary to assemble a training regimen that considers exercise time. It is therefore necessary for future investigations to further examine the duration, intensity and frequency aspects of EEs that do not affect the effectiveness of resistance exercise.

## Conclusions

EE increased the weight of hypertrophic muscle caused by functional OL regardless of exercise duration. However, exercise duration did not affect mTOR signaling. In contrast, long‐term EE promoted mitochondrial biogenesis even during muscle hypertrophy and increased protein degradation, oxidative stress and ubiquitin, which may suppress muscle hypertrophy.

## Conflict of interest

The authors declare no conflict of interest.

## Author contributions

TS, TO and TT conceived and designed the project; TS and TO performed the experiments; TS analyzed the data; TS wrote the manuscript and revisions, which were checked by TT; all authors read and approved the final manuscript.

## Data Availability

Data will be available from the corresponding author upon reasonable request.
